# Splice variants of the endonucleases XPF and XPG contain residual DNA repair capabilities and could be a valuable tool for personalized medicine

**DOI:** 10.18632/oncotarget.23105

**Published:** 2017-12-08

**Authors:** Janin Lehmann, Steffen Schubert, Christina Seebode, Antje Apel, Andreas Ohlenbusch, Steffen Emmert

**Affiliations:** ^1^ Clinic and Policlinic for Dermatology and Venereology, University Medical Center Rostock, Rostock, Germany; ^2^ Information Network of Departments of Dermatology (IVDK), University Medical Center Goettingen, Goettingen, Germany; ^3^ Department of Dermatology, Venereology and Allergology, University Medical Center Goettingen, Goettingen, Germany; ^4^ Department of Pediatrics and Adolescent Medicine, Division of Pediatric Neurology, University Medical Center Goettingen, Goettingen, Germany

**Keywords:** xeroderma pigmentosum, nucleotide excision repair, interstrand crosslink repair, spontaneous mRNA splice variants

## Abstract

The two endonucleases XPF and XPG are essentially involved in nucleotide excision repair (NER) and interstrand crosslink (ICL) repair. Defects in these two proteins result in severe diseases like xeroderma pigmentosum (XP). We applied our newly CRISPR/Cas9 generated human *XPF* knockout cell line with complete loss of XPF and primary fibroblasts from an XP-G patient (XP20BE) to analyze until now uncharacterized spontaneous mRNA splice variants of these two endonucleases. Functional analyses of these variants were performed using luciferase-based reporter gene assays. Two *XPF* and *XPG* splice variants with residual repair capabilities in NER, as well as ICL repair could be identified. Almost all variants are severely C-terminally truncated and lack important protein-protein interaction domains. Interestingly, XPF-202, differing to XPF-003 in the first 12 amino acids only, had no repair capability at all, suggesting an important role of this region during DNA repair, potentially concerning protein-protein interaction. We also identified splice variants of *XPF* and *XPG* exerting inhibitory effects on NER. Moreover, we showed that the XPF and XPG splice variants presented with different inter-individual expression patterns in healthy donors, as well as in various tissues. With regard to their residual repair capability and dominant-negative effects, functionally relevant spontaneous *XPF* and *XPG* splice variants present promising prognostic marker candidates for individual cancer risk, disease outcome, or therapeutic success. This merits further investigations, large association studies, and translational research within clinical trials in the future.

## INTRODUCTION

The nucleotide excision repair (NER) pathway is a central DNA repair mechanism eliminating a variety of bulky DNA lesions like pyrimidine photo products [1]. If these sorts of lesions accumulate in the genome, this can result in a cancer prone mutator phenotype as seen in the autosomal recessive model disease xeroderma pigmentosum (XP). Patients present with a high frequency of UV-induced skin tumors as well as a reduced life span [2]. According to the respective disease-causing defective genes (*XPA-XPG*) seven XP complementation groups (XP-A to XP-G) and a variant form with a mutated translesion polymerase (*PolH* gene) have been identified so far [3].

NER is characterized by subsequent steps and involves more than 30 proteins. Damage detection can be subdivided into global genome repair (GGR), via the XPC-hHR23B-centrin2 complex together with the DNA damage binding complex (XPE), and transcription coupled repair (TCR), initiated by a stalled RNA polymerase II and transcription factor IIH (TFIIH). Damage demarcation is performed by XPA and XPD followed by DNA unwinding around the damage catalyzed by the ATPase (XPB) and helicase (XPD) activities of TFIIH. The damage is excised by two subsequent incisions performed by XPF/ERCC1 and XPG 5’ and 3’ to the DNA lesion releasing the damage containing oligonucleotide (24–32 nts) from the DNA double helix. Finally, the gap is filled by DNA polymerases (δ, ε, or κ) using the undamaged strand as a template and nick sealing by DNA ligases (I or III) [1]. Furthermore, the endonuclease complex XPF/ERCC1 is also implicated in interstrand cross-link (ICL) repair [4]. In addition, it is known that decreased NER levels are a risk factor for several cancer and tumor entities in the normal population [5]. An implication of the *XPF* and *XPG* genes as promising prognostic marker for skin cancer risk as well as for disease outcome is based on genome-wide association studies of single nucleotide polymorphisms (SNPs) and protein expression analyses [6–8].

The human genes for the endonucleases *XPF* (ERCC4) (OMIM: 278760) and *XPG* (ERCC5) (OMIM: 278780) encode for 916 and 1186 amino acid (aa) proteins cleaving 5’ and 3’ of UV induced lesions. They are located on chromosomes 16p13.2-p13.1 and 13q32.3-q33.1 [9, 10]. It has been demonstrated that these essential repair genes show a high number of physiologically occurring spontaneous alternatively spliced transcripts with yet unknown functions [11]. Furthermore, the differential expression level of splice variants in several tissues has been shown to be more suitable to distinguish between oncogene and non-oncogene samples than the primary gene transcript itself [12].

In this study we focus on the catalytically active subunits of the NER endonucleases, XPF and XPG. It has been difficult in the past to study the functions of the XPF/ERCC1 complex so far, as there was no appropriate patient cell line without residual repair capability as seen in the XP-G patient XP20BE [13]. We decided to use primary patient fibroblast with a transient transfection approach, as there are no immortalized XP-G patient cells without residual alleles or repair capabilities. Recently, we created a complete *XPF* knockout (KO) via the CRISPR/Cas9 system [14]. We now demonstrate that there are several spontaneous *XPF* and *XPG* mRNA splice variants with residual repair capability and/or inhibitory effects on NER as well as ICL repair in these cells. These XPG splice variants show expression differences in regard to various tissues and are differentially expressed among individuals. Therefore, the variants may present promising prognostic marker candidates for individual cancer risk, disease outcome, or therapeutic success.

## RESULTS

### Protein expression and subcellular localization of *XPF* and *XPG* splice variants

**Figure 1:**
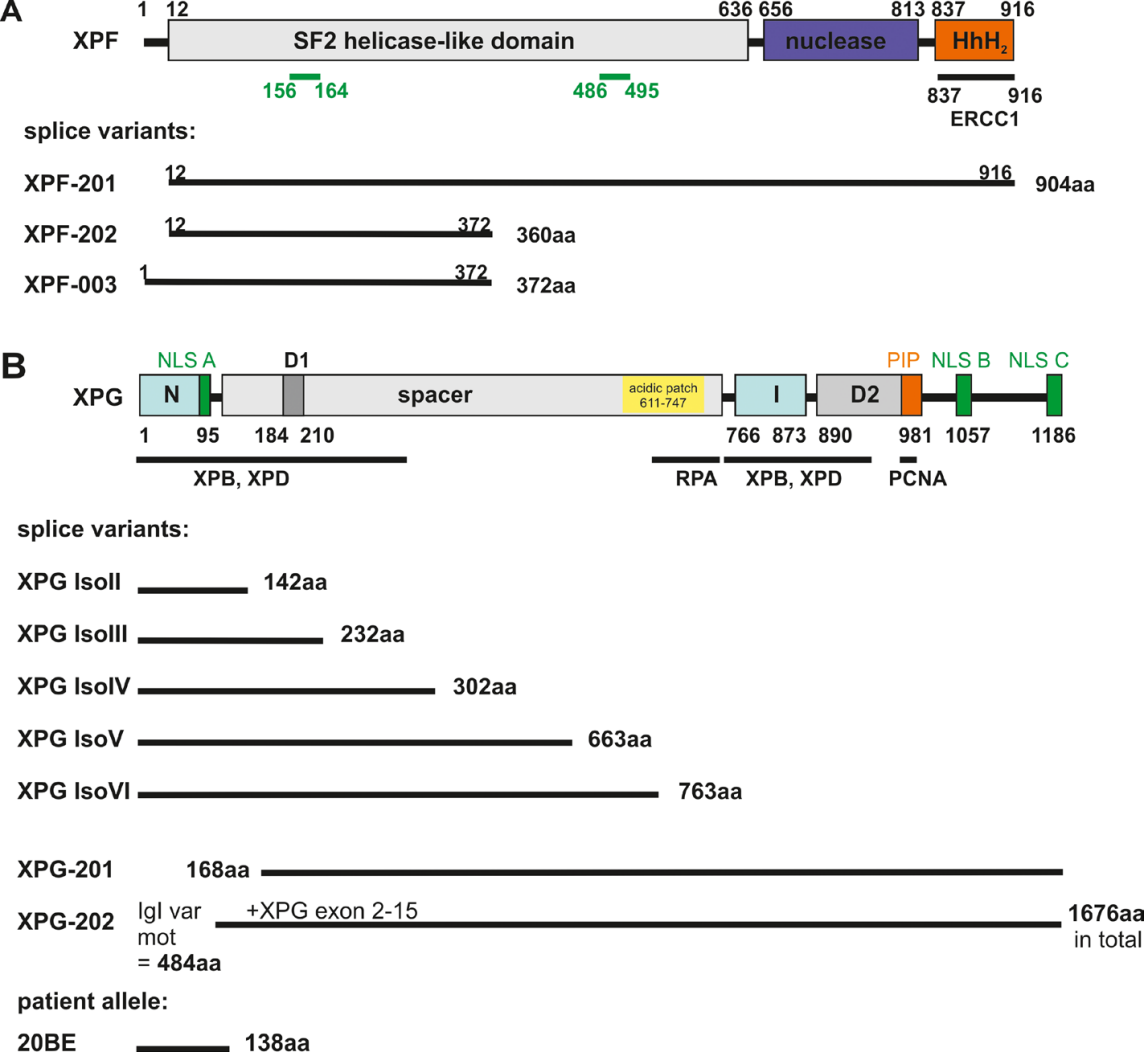
Schematic overview of XPF/XPG domains and their spontaneously and physiologically occurring mRNA splice variants The colored bars show functional protein domains (e.g. nuclease domain (blue), Helix-hairpin-Helix (HhH)_2_ motif (orange)). Putative NLS are depicted in green and black lines highlight protein-protein interaction domains. (**A**) The *XPF* gene undergoes alternative splicing resulting in C- and/or N-terminally truncated physiologically occurring variants. The isoform XPF-201 only lacks the first 12 aa, while XPF-202 and XPF-003 are severely C-terminally truncated. These two variants lack functional domains, e.g. the nuclease domain. (**B**) The N and I domains (light blue-green) that form the catalytic center of the XPG endonuclease are separated by a spacer region in the primary sequence, after protein folding they come close together. XPG also has several C-terminally truncated variants (XPG IsoII - IsoVI) missing one or more of the functional domains. On the other hand, variant XPG-201 misses exons one - four (in frame) including one of the nuclease domains (N) and part of an interaction patch with TFIIH. XPG-202 only lacks exon one, but in addition contains the immunoglobulin like variant motif of 484 aa, caused by splicing defects leading to a conjoined gene. The XP20BE patient allele is severely truncated and patient primary fibroblasts were used for functional testing. Modified from [10, 11, 71–74].

**Figure 2:**
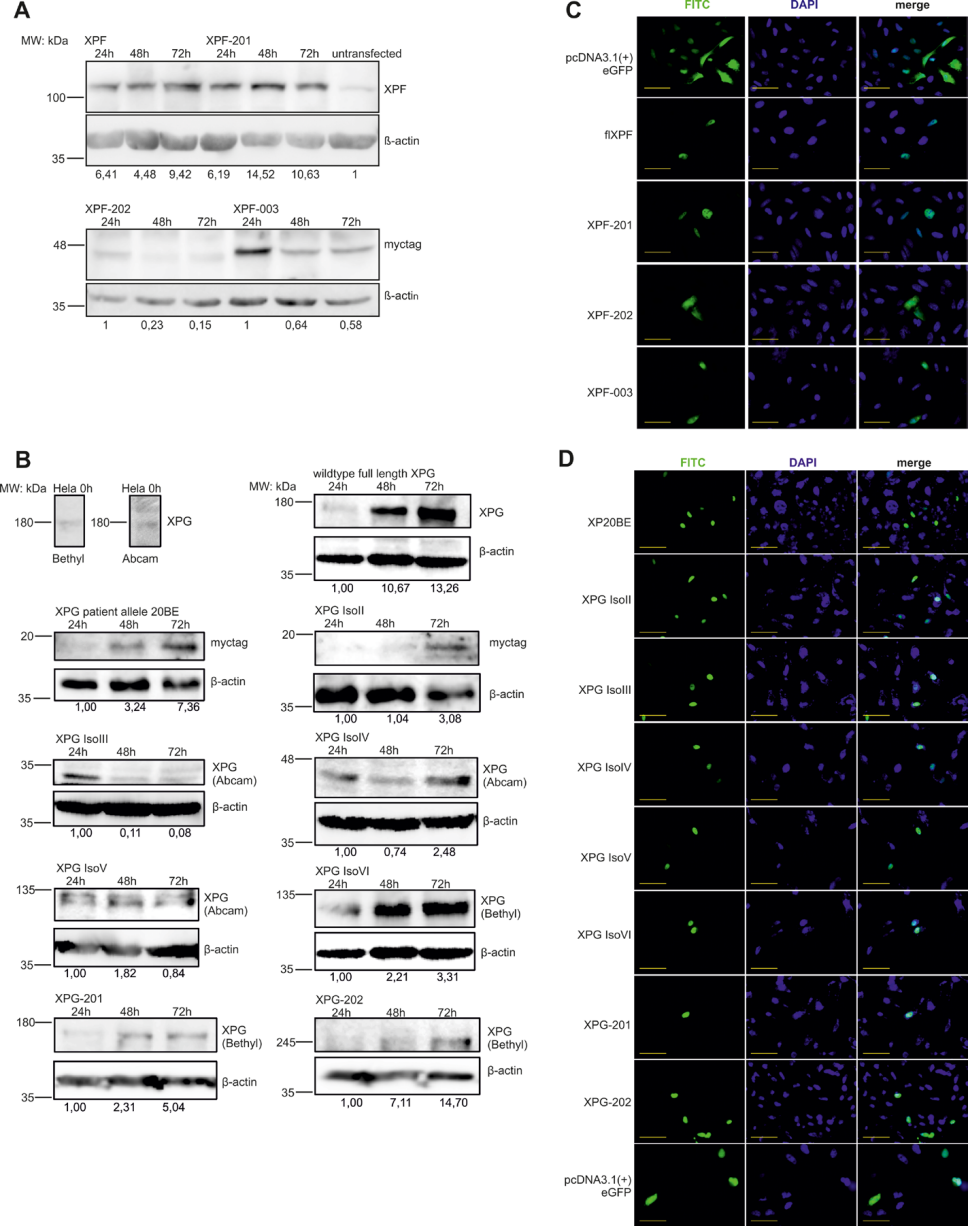
Protein levels of *XPF* and *XPG* splice variants over time and subcellular localization of eGFP-tagged isoforms and the XP-G patient allele To check transfection and overexpression of the splice variants, protein levels in HeLa cells were determined using horizontal SDS Page followed by immunoblotting. (**A** and **B**) Cells were transiently transfected with the different constructs harvested after 24 h, 48 h and 72 h, and stained with an anti-XPF/XPG or an antibody directed against the myc-tag. Anti-β-actin staining was used for normalization. Protein levels were quantified in regard to untransfected control cells or to the 24 h value in the case of the myc-tagged constructs as there is no endogenous myc-tag protein. One of three representative experiments is shown. (**C** and **D**) For subcellular localization XPF and XPG isoforms as well as the XP20BE patient allele were cloned into an pcDNA3.1(+)eGFP expression vector and overexpressed in HeLa cells for 48 h. Additionally, DAPI staining was performed to visualize the nucleus. Scale bar = 50 µm.

All until now known, physiologically occurring spontaneous mRNA splice variants (Figure 1A–1B) of the *XPF* (XPF-201, XPF-003, XPF-202) (ENA numbers LT827130-LT827133) and *XPG* (XPG IsoII, XPG IsoIII, XPG IsoIV, XPG IsoV, XPG IsoVI, XPG-201, XPG-202) (ENA numbers LM994821, LM994822, GenBank numbers AH009656, AF255431-AF255442) genes were amplified from wildtype (WT) primary fibroblast or MRC5Vi cDNA, transcribed from mRNA, and cloned into different expression plasmids: pcDNA3.1 (+), pcDNA3.1 (-)mycHisA2, and pcDNA3.1 (+)eGFP (C-terminal tag). All expression cassettes have been validated by Sanger sequence analyses. Figure 1A and 1B illustrate an overview of all *XPF* and *XPG* splice variants on protein level depicting domains lost due to truncating mutations. With the aim to analyze transient protein expression, we performed immune blot analyses of the different recombinant XPF and XPG constructs over 72h (Figure 2A–2B). It is shown that all *XPF and XPG* isoforms were successfully overexpressed over time and resulted in proteins of the calculated size in the case of XPF and its variants. Whilst WT XPF levels peak at 72 h, its isoform XPF-201 shows highest protein levels after 48h. Interestingly, protein levels of the shorter isoforms XPF-202 and XPF-003 decrease over time. WT XPG, XPG IsoII, XPG IsoIV, XPG IsoVI, XPG-201 and XPG-202 as well as the XP20BE patient allele show an increase in expression within the time scale, only expression of XPG IsoIII decreases over time. Only XPG IsoIV presents with a band at the expected size of 35kDa, while XPG IsoV, XPG IsoVI, XPG-201, and XPG-202 travel between 50 and 60 kDa above their expected size.

To investigate nuclear localization of the splice variants, the compartment where DNA repair takes place, GFP-fusion proteins of the splice variants were produced in HeLa cells and evaluated by fluorescence microscopy. 48h after overexpression, all *XPF* and *XPG* isoforms as well as the WT proteins, could be found in the nucleus, whereas XPF-202 showed a diffuse distribution within the cell (Figure 2C–2D). Theoretically, all isoforms should be able to participate in different DNA repair pathways based on stable protein expression and nuclear localization if they had residual enzymatic or structural functions.

### The involvement of *XPF* and *XPG* splice variants in NER and ICL repair

**Figure 3:**
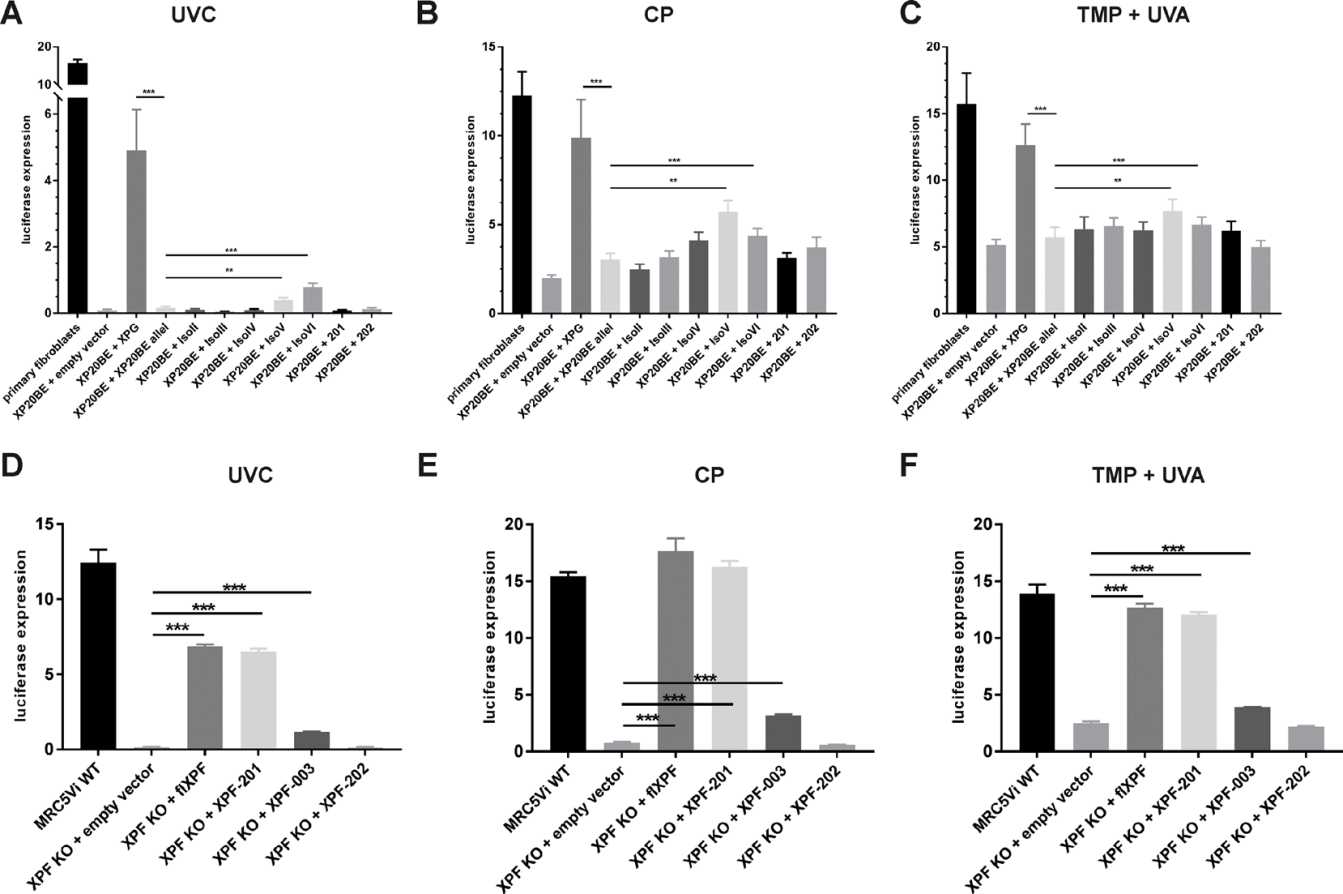
Reactivation of a reporter gene after treatment with UVC, cisplatin, or trimethylpsoralen activated by UVA light in *XPF* KO cells or XP-G patient cells complemented with *XPF* or *XPG* splice variants Firefly plasmids were treated with (**A** and **D**) UVC irradiation, (**B** and **E**) cisplatin (intrastrand crosslinks), or (**C** and **F**) trimethylpsoralen activated by 1 J/cm^2^ UVA irradiation (interstrand crosslinks), to induce specific lesions, transfected into MRC5Vi WT/XP20BE primary fibroblasts (XP-G patient cells) or *XPF* KO cells, and complemented with plasmids coding for full-length proteins and the different splice variants. The relative repair capability is calculated as the percentage (luciferase expression) of the reporter gene activity (firefly luciferase) compared to the untreated plasmid, after normalization to an internal co-transfected control (*Renilla* luciferase). Data are presented as the mean ± SEM. The one-tailed, unpaired student’s *t*-test was applied, ^***^ *P* < 0.001 or ^**^ *P* < 0.01. At least four independent experiments in triplicates were performed.

As illustrated in Figure 1, *XPF* and *XPG* isoforms either show C-or N-terminal truncations, leading to full or partial loss of protein domains, which are important for enzymatic activity or protein-protein interactions. We applied the functional Host Cell Reactivation (HCR) assay to investigate residual repair capability. The assay was originally applied to study the repair of UV induced DNA lesions (6–4photoproducts (6–4PPs) and cyclobutane pyrimidine dimers (CPDs)) repaired by NER. We also adapted the assay to the repair of intrastrand and interstrand crosslinks by treating the firefly reporter plasmid with *cis*-diammineplatinum (II) dichloride (CP) and 4,5’,8-trimethylpsoralen (TMP) plus UVA light as described in Lehmann *et al.* 2017 [14]. NER and ICL repair activity of all *XPG* (in XP20BE cells) and XPF isoforms (in MRC5Vi XPF KO Cells) was determined (Figure 3A–3F) in comparison to primary WT fibroblasts and WT MRC5Vi cells. Primary WT fibroblasts exhibited a NER repair capability of 15% (15.72 ± 0.96%), while XP20BE patient cells show no residual repair (0.09% ± 0.03) (see Figure 3A). This effect could only be rescued by complementation with full-length XPG (flXPG) or *XPG* isoforms V and VI in comparison to the empty vector control (XPG: 4.9 ± 1.22%, IsoV: 0.40 ± 0.07%, IsoVI: 0.78 ± 0.12%) close to WT levels of repair (15.72 ± 0.96%). For ICL repair, WT repair levels of about 15% (CP: 13.92 ± 1.71%, TMP + UVA: 17.25 ± 3.34%) were measured (Figure 3B–3C). Interestingly, co-transfection with fl*XPG* was closer to WT levels than observed for UVC induced lesions (CP: 11.61 ± 2.75%, TMP + UVA: 12.71 ± 1.55%). Again, isoform V and VI showed a significant increase in repair capability (IsoV: CP 5.98 ± 0.98%, TMP + UVA 8.55 ± 1.54% and IsoVI: CP 4.57 ± 0.76%, TMP + UVA 9.94 ± 2.32%), while the other isoforms did not have a significant effect. Overall basic levels of repair were higher than for UVC (CP 1.98 ± 0.19%, TMP + UVA 5.12 ± 0.43%), suggesting that the involvement of XPG is more important in NER than ICL repair.

Residual repair capabilities of *XPF* splice variants were studied in the same way in the complete *XPF* KO [14] cells compared to MRC5Vi WT cells (12.45 ± 0.86%). *XPF* KO cells show a nearly complete loss of repair capabilities (0.15 ± 0.02%), but repair can be rescued by complementation with full-length *XPF* (6.87 ± 0.13%) (Figure 3D–3F). Two *XPF* splice variants exhibited significant residual repair capabilities in NER as well as ICL repair (XPF-201: UVC 6.50 ± 0.22%, CP 16.28 ± 0.51%, TMP + UVA 12.06 ± 0.22% and XPF-003: UVC 1.15 ± 0.02%, CP 3.17 ± 0.10%, TMP + UVA 3.89 ± 0.03%). As shown in Figure 1B XPF-201 only lacks the first 12 aa of the protein, whereas XPF-003 is severely C-terminally truncated, lacking the endonuclease domain. Interestingly, XPF-202, another variant, that only differs from XPF-003 in the first 12 aa did not display any significant repair capability at all (UVC 0.15 ± 0.02%, CP 0.58 ± 0.03%, TMP + UVA 2.17 ± 0.06%) (Figure 3D–3F). This prompts towards a high importance of those 12 N-terminal aa of the XPF protein, e.g. due to an undescribed essential interaction domain of the protein, which may be necessary for structural or catalytic roles of the protein.

### *XPG* and *XPF* splice variants exhibit inhibitory effects on NER

**Figure 4:**
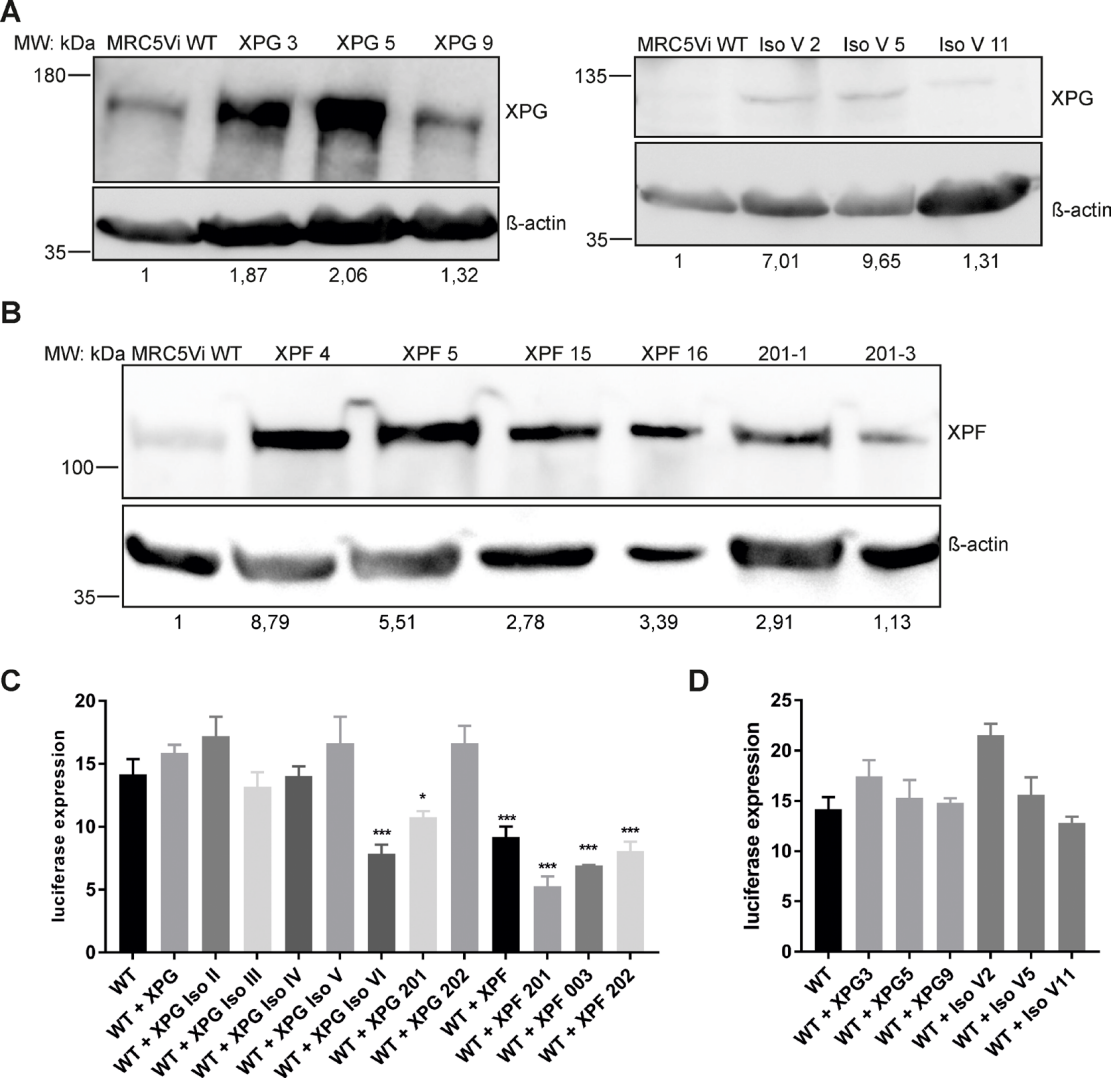
Inhibitory effects of the overexpression of *XPG*, *XPF*, or respective splice variants in MRC5Vi WT cells Immunoblot analyses of single clones and reactivation of a reporter gene after treatment with UVC in MRC5Vi WT cells and single clones. Antibodies for (**A**) XPG or (**B**) XPF were used and β-actin staining was applied for normalization. Representatively, one of three independent experiments is shown. Protein levels in MRC5Vi WT cells were set to one. (**C** and **D**) For functional analyses, firefly plasmids were treated with UVC irradiation, transfected into MRC5Vi WT or single clones overexpressing XPG, XPF, or respective splice variants. The relative repair capability is calculated as the percentage (luciferase expression) of the reporter gene activity (firefly luciferase) compared to the untreated plasmid, after normalization to an internal co-transfected control (*Renilla* luciferase). Data are presented as the mean ± SEM. The one-tailed, unpaired student’s *t*-test was applied; significances are displayed with regard to WT cell repair capability, ^*^ *P* < 0.05 or ^***^ *P* < 0.001. At least four independent experiments in triplicates were performed.

In order to investigate the interference of *XPG* and *XPF* spontaneous mRNA splice variants with endogenous DNA repair, exemplarily NER, cell lines stably overexpressing these variants were generated in MRC5Vi WT cells. To test the effect on endogenous NER ability of MRC5Vi WT cells, three positive clones with a similar level of overexpression were selected for each variant or full-length protein, and repair capabilities were then averaged for statistical purposes. Figure 4A and 4B exemplarily show the stable overexpression of the full-length proteins (XPG 180kDa, XPF 112kDa) as well as their splice variants (XPG IsoV 100kDa, XPF-201 112kDa) in MRC5Vi WT cells, displaying levels of overexpression between 1.13–9.65 fold. XPG-201, a variant N-terminally lacking the first four exons in frame resulting in a loss of part of XPG’s endonuclease domains (N-domain), showed a significant dominant negative effect on NER (10.75 ± 0.50%) compared to MRC5Vi WT cells (14.17 ± 1.20%) (see Figure 4C). The same effect could be observed for XPG IsoVI, the variant exhibiting the highest residual repair capability in XPG deficient cells, lacking the endonucleolytic I-domain (7.85 ± 0.72%). In turn, all three *XPF* splice variants, as well as the full-length protein, significantly reduced repair capabilities of MRC5Vi WT cells (XPF: 9.19 ± 0.83%, XPF-201: 5.26 ± 0.80%, XPF-003: 6.90 ± 0.06%, XPF-202: 8.08 ± 0.73%). Figure 4D shows that individual clones with different levels of overexpression have a different effect on influencing repair capability. Anyhow, the average of three clones presents a balanced and averaged measurement for inhibitory effects. In summary, two *XPG* splice variants (XPG IsoVI and XPG-201) as well as WT *XPF* and all three splice variants (XPF-201, XPF-003, XPF-202) inhibited the endogenous NER capabilities of MRC5Vi WT cells.

### Differential expression of functionally relevant *XPG* splice variants in tissues and healthy individuals

**Figure 5:**
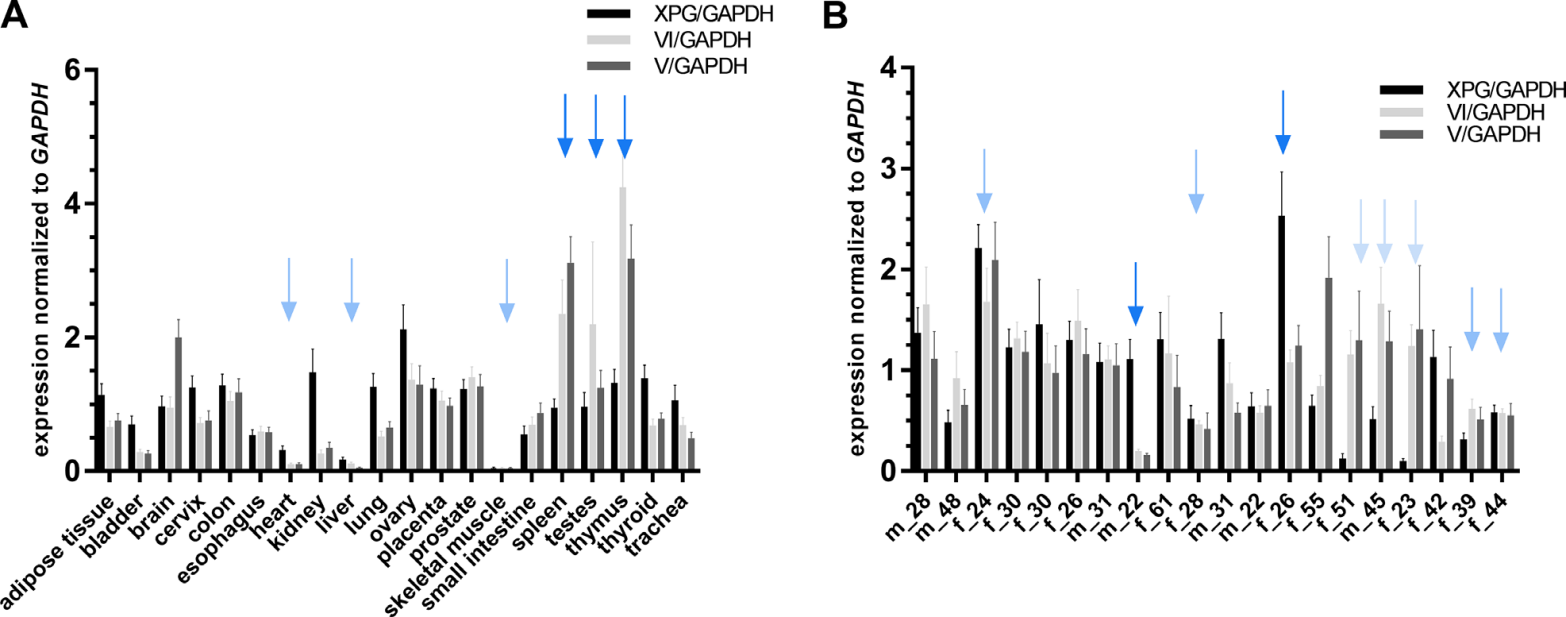
Analysis of *XPG* expression in different tissues and human blood samples RNA samples from 20 tissues (**A**) and 20 unrelated human individuals (**B**) were transcribed into cDNA and subject to qRT-PCR measurements. Expression levels are shown as absolute values in relation to *GAPDH* used as the internal control to normalize the expression of the target gene or isoform. Results are shown as mean values from three independent experiments in duplicates. Data are presented as mean ± SEM. Colored arrows indicate groups with similar expression patterns.

The physiological relevance of *XPG* isoforms with repair catalyzing functions (isoform V and VI), was analyzed via detection of specific mRNA expression in 20 different healthy human tissues. Unfortunately, the material was only sufficient to analyze *XPG*, but not *XPF*, variants. Figure 5A shows the comparison of the three *XPG* species in the 20 different tissues. WT *XPG*, as well as both functionally relevant isoforms were expressed in all tissues. Heart, liver and skeletal muscle exhibited very low expression levels of all three *XPG* transcripts (light blue arrows), while the expression of both isoforms, V and VI, was high in spleen, testes, and thymus compared to the primary transcript (dark blue arrows). Furthermore, to compare human inter-individual expression levels of the three *XPG* transcripts, blood samples from 20 healthy donors (age 22–61, 7 male and 13 female) were compared (see Figure 5B). We could identify three different patterns. The expression profile either displayed high or low expression of all three transcripts (middle blue arrows), expression of a predominant primary transcript (dark blue arrows), or predominance of the two isoforms V and VI (light blue arrows). All in all, isoform VI, containing the highest residual catalytic activity in the functional experiments, was predominantly expressed in blood samples of 80% of the investigated subjects. Relations of expression between age and sex could not be observed in this small collective.

## DISCUSSION

The two endonucleases XPG and XPF are involved in several essential cellular processes like NER and ICL repair, in addition to basal transcription. They exert catalytic as well as structural functions. Mutations in these two genes result in complex genotype-phenotype correlations. Notably, patients with pathological *XPG* splice variants that still contain residual functions show a milder form of the XP/Cockayne syndrome (CS) complex phenotype and a longer skin cancer free survival time [10]. More precisely, a splice variant of a 28-year-old patient with minimal residual function has led to a meaningful prolongation of his life [15]. On the other hand, a dominant negative splice variant of an XP-C patient, that was still functionally active, reduced NER activity by approximately 50% [16]. Eventually, this variant was identified in about 40% of normal individuals and could be associated with a two-fold increased melanoma risk [17].

### Cloned *XPF* and *XPG* splice variants produce stable proteins and localize to the nucleus

A prerequisite for analyses of *XPF* and *XPG* splice variants was to generate artificial constructs for further functional *in vitro* experiments. The generated constructs lead to stable overexpression of proteins (see Figure 2A–2B). In the case of XPF, protein products of the expected size were produced. XPG shows a band around 180kDa, which is above the calculated size of 136kDa, caused by its acidic patch in the spacer region (amino acids 611–747, see Figure 1B). Due to an abnormal mass-to-charge ratio the migration behavior in SDS-Page is changed [18–20]. This is also the case for the larger splice variants (XPF IsoV-VI, XPG-201, XPG-202). The acidic patch is not present in the rudimentary patient allele 20BE or in the short XPG IsoII, but they do not travel according to their size due to the myc/HisA2-tag, which is positively charged and binds more than the statistical amount of SDS. Additionally, the splice variants exhibit different peak protein level over time. These differences in expression could be explained by proteolytic degradation [21] or triggered by toxic effects of shorter isoforms possibly constituting inhibitory effects on DNA repair.

The nucleus is the cellular compartment where DNA repair takes place making it necessary that all proteins involved are either passively or actively imported. Some of the truncated splice variants (XPF-003 and XPF-202, as well as XPG II-VI) lack the C-terminal nuclear localization signal (NLS) (XPF aa486–495, XPG aa1057–1074 and aa1171–1185) (see Figure 1A). The smaller variants (XPG-II-IV) should be able to passively diffuse into the nucleus due to their size, whereas XPF-003, XPF-202, XPG-V, XPG-VI, XPG-201, and XPG-202 depend on a NLS, suggesting a less efficient transport for these variants as they only contain one of two (XPF) or three (XPG) NLS. Fluorescent analysis (see Figure 2C–2D) showed that after 48 h all isoforms had been imported to the nucleus, meaning that the activity of one NLS is sufficient and apparently a prerequisite for catalytic activity of the splice variants. Only XPF-202 displayed a rather diffuse cellular distribution with an accumulation in the nucleus. As seen in an XFE patient, dysfunctional or unstable XPF proteins exhibit an abnormal subcellular localization, which may indicate that XPF-202 is not involved in functional DNA repair [22, 23] (see Figure 3D–3F).

### Splice variants of the two endonucleases *XPF* and *XPG* show residual repair capabilities in NER and ICL repair

Physiologically occurring, spontaneous mRNA splice variants may contain residual catalytic or putative structural DNA repair functions. *XPG* splice variants II-VI lack the endonuclease activity containing I region (see Figure 1), anyhow XPG IsoV and VI contain residual repair capabilities as well as two *XPF* splice variants (XPF-201 and XPF-003). Interestingly, the isoform XPF-202 that only differs from XPF-003 in the first 12 aa did not show any complementation.

Complementation of XPG- or XPF-deficient cells with full-length proteins only resulted in partial restoration of repair capability (XPG 4.90%, XPF 6.87%) in comparison to WT levels (primary fibroblasts 15.72%, MRC5Vi WT 12.45%), caused by transfection efficiencies of only 50%. Moreover, both proteins are involved in basal transcription making the HCR assay a qualitative rather than quantitative read-out of repair capability. Different damage loads can be excluded in this experimental setting due to the fact that i) the level of WT MRC5Vi cells is comparable for all three lesions, as well as for WT fibroblasts; ii) the results are always presented as percent expression of untreated vector within one group of DNA damages, and iii) a batch treatment of plasmids was done and used for all respective experiments. Splice variants do even exhibit a smaller complementation effect, *XPG* isoform V and VI as well as XPF-003 only elicit minor relative increases in NER and ICL repair capability, yet, clearly statistically significant in comparison to mock vector transfectants (see Figure 3). Initially this seems to be a marginal increase, but regarding a patient’s survival or skin cancer free life period the effect might be tremendous. As mentioned before, these isoforms contain only one NLS, decreasing nuclear import activity, explaining the low increase in repair capability in partial. A stronger effect might be observable if these variants could be transported into the nucleus more efficiently. In a patient with nearly normal *XPG* mRNA expression Emmert *et al.* found one allele with an early stop codon mutation, inherited from her father, while the maternal allele only showed a single base missense mutation. This resulted in a residual repair activity of 10% and the 14 year old Caucasian girl (XP65BE) presented with a mild phenotype displaying sun sensitivity, no skin cancer, and no neurological symptoms [24]. This implicates that minor repair activities of splice variants may have a huge influence on the cell’s repair capabilities and therefore could be accounted for mild clinical features and strong influence on the severity of disease progression as well as skin cancer free survival time.

Unfortunately, the mechanism of mammalian ICL repair is not completely understood, yet. As unhooking is a key step during ICL repair, roles of the two endonucleases XPG and XPF/ERCC1 are of special interest. It depends on the position of an ICL whether XPF/ERCC1 is able to cleave on either side [25], but also XPG may be involved, cutting 3′ to a junction between duplex and single-stranded DNA [26]. Notably, XPG does not seem to be essential for ICL repair in contrast to XPF (see Figure 3B–3C) [27]. As previously shown, human cells deficient in XPG presented unhooking kinetics of psoralen + UVA induced ICLs close to WT levels, while XPF/ERCC1 deficient cells were not able to unhook these lesions [4, 28, 29].

This is in concurrence with our results that propose a fundamental role of the XPF/ERCC1 complex in ICL repair, as we could nearly detect no residual repair capability in our *XPF* KO cells (see Figure 3E–3F). XPF/ERCC1 is known to interact with the scaffold protein SLX4 and two other structure specific endonucleases Mus81/Eme1 and SLX1, and SLX4 is thought to enhance XPF’s endonuclease activity [30–32]. The interaction between SLX4 and XPF/ERCC1 seems to be important outside NER, as an siRNA knockdown of SLX4 rendered cells sensitive to crosslinking agents, but not UV irradiation [32]. Our results suggest an important protein interaction domain between the very N-terminal part of the XPF protein and SLX4, as XPF-003 showed residual repair capability in contrast to XPF-202 (see Figure 3D–3F). In regard thereto, a double knockout of *XPF* and *SLX4* is of special importance to test the complementation ability of the XPF-003 splice variant. This double knockout should eradicate XPF-003’s complementation ability most importantly in regard to CP and TMP + UVA induced lesions, indicating dependence on the presence of SLX4. Moreover, XPF-202 showed a diffuse cellular localization and no clear nuclear accumulation (see Figure 2D). A disrupted interaction with SLX4, an important platform and scaffold protein to organize DNA repair factors, could also be an explanation for this. It is supposed that XPF/ERCC1 and SLX4 simultaneously load onto the ICL [33] and SLX4 also functions as a SUMO E3 ligase sumoylating itself, as well as XPF [34]. Furthermore, the XPF/ERCC1 endonuclease is also important for recombination and DSB repair [35–38] meriting further investigations to discover whether the interaction between XPF and SLX4 is important for unhooking or other crosslink repair associated pathways. Another important interaction factor is the single stranded DNA binding protein RPA. It is essential for ICL unhooking, by permitting XPF/ERCC1 to overcome inhibitory structures at ICLs, like the nascent leading strand [27]. So far only one essential residue for interaction between XPF/ERCC1 and RPA has been identified in the afore mentioned XFE patient [39], but there might also be other important residues, possibly in the first 12aa of the protein.

Commonly, all functionally active splice variants of *XPF* and *XPG* have evolved from alternative splicing and intron retention [11]. Intron gain or loss has been an important evolutionary engine over time [40]. In the literature, it is postulated that intron-retaining splice variants of transcription factors could be risk factors for various cancer entities, e.g. breast cancer, lymphoma or melanoma [41–43].

Most importantly, it remains to be elucidated how splice variants exert residual repair capabilities as *XPG* IsoV, VI, and XPF-003 lack at least parts of the endonuclease domains (see Figure 1). In addition to protein-protein interactions with other repair factors the existence of a cellular backup mechanism for the XPG endonuclease has been suggested demonstrating that endonuclease defective XPG was able to perform accurate NER in living cells [44]. It can be proposed that severely truncated *XPG* splice variants can structurally complement XPG-defective cells. This would result in functional NER by recruitment of other structure-specific endonucleases like e.g. Exo1 or Fen1, cleaving the DNA allowing the repair to proceed [45, 46]. It merits further investigations to prove this hypothesis. For example, recruitment of the XPF/ERCC1 complex to DNA damage in XPG-deficient cells expressing the truncated splice variants would be of special interest.

### *XPG* and *XPF* splice variants exert a dominant negative effect on wild type NER capacities

We characterized the interference of splice variants with endogenous DNA repair to investigate dominant negative inhibition. Thereby, we identified two *XPG* splice variants (XPG IsoVI and XPG-201), as well as WT *XPF* and all three splice variants (XPF-201, XPF-003, XPF-202), to exhibit a dominant negative effect on MRC5Vi WT NER capabilities (see Figure 4C–4D). A larger part of protein-encoding genes is subject to physiological splicing, but pathological splicing has been reported in cancer tissue [47, 48]. Alternative splicing could function as a mechanism for repression of WT protein function, e.g. *Chk2* splice variants have an effect on downstream substrates like p53, and Cdc25A/C involved in cell cycle arrest and apoptosis [49]. It has already been shown that a polymorphism of the splice acceptor site in the *XPC* gene leads to the expression of a functionally inhibitory splice variant reducing NER by about 50% [16]. It occurs as a haplotype with a second polymorphism in the *XPC* gene [50] that is associated with an increased risk for skin cancer development, SCCs and melanoma [17, 51]. Splice variants of the telomerase catalytic subunit (hTERT) lacking telomerase activity, due to missing parts of the catalytic core, can inhibit endogenous telomerase activity in developing tissues, resulting in telomere shortening and chromosome end-to-end fusions [52, 53]. In addition, dominant negative effects are often observed for splice variants of multisubunit proteins, e.g. isoleucyl-tRNA synthetase. Co-expression of the variant and the endogenous WT protein may result in competition for functional complex formation due to dysfunctional complexes in need of the same binding partners [54]. We would expect a similar mechanism behind the inhibitory effects of XPF and its splice variants in view of heterodimer formation with ERCC1. However, the interaction between ERCC1 and XPF is conferred by the (HhH)_2_ motif, which is only present in XPF-201, as XPF-202 and XPF-003 are severely C-terminally truncated.

Another possible explanation might be protein overload in the cell, due to the strong and artificial overexpression, resulting in unproductive protein interactions of splice variants with residual repair capability. Moreover, protein ratios of XPG/XPF could be disturbed. The dual incision process is highly coordinated between XPF/ERCC1 and XPG in subsequent binding and cutting steps and might be sensitive to changes in molecular ratios.

### Implication of *XPG* isoforms for personalized medicine and further perspectives

We were able to detect differential patterns of three functional XPG transcripts between individuals as well as different tissues (see Figure 5A–5B). As mentioned before, expression levels of splice variants have been shown to be more suitable to distinguish between oncogene and non-oncogene samples than the primary gene transcript itself [12]. This could be an indicator for the existence of an individualized overall repair capacity that might even differ between a person’s tissues. Therefore, the expression of XP splice variants could determine a patient’s resistance to different therapies, e.g. in chemotherapy against cancer. A weak chemotherapeutic response could be explained by different levels of DNA repair and has already been shown to be a target for small molecule inhibitors [55]. Additionally, it is known that resistances to platinum-based therapy correlate with high expression of ERCC1 [56] and can be reversed by blocking the interaction between ERCC1 and XPA, which is essential for NER, sensitizing cancer cells to NER substrates. Furthermore, inhibitors disrupting the XPF-ERCC1 interaction have been identified in *in silico* drug screens [57]. High expression of XP genes, like *ERCC1*, has also been correlated with poor therapy response in different cancer entities like e.g. non-small cell lung cancer, esophageal cancer, breast cancer, colorectal cancer, as well as head and neck cancer [58–61]. Testes tumors with reduced levels of NER proteins could be cured after application of a cisplatin-based therapy, even though they were already in an advanced metastasizing state. Meaning that low expression of XPF and XPG is beneficial for patients with advanced testes tumors in regard to therapeutic responses [62]. Interestingly, in our expression analyses of 20 different tissues we found that both XPG isoforms, V and VI, were surpassingly high in testes compared to WT XPG levels.

Anyhow, tumor-specific splice variants often tend to be overexpressed [63–65]. In order to apply splice variants as prognostic marker, residual repair capabilities and duration of skin-cancer free survival time in patients missing the primary transcript [15] as well as dominant negative functions in healthy individuals have to be balanced and evaluated in larger collectives. The HCR assay has already been used in molecular epidemiology studies to correlate low NER function in peripheral blood lymphocytes with an increased risk of cancer [66]. Furthermore, Wei et al. showed decades ago that a reduced NER capacity as assessed with HCR and peripheral blood lymphocytes is an independent melanoma and basal cell carcinoma risk factor [67, 68].

Our small collective of 20 healthy donors as well as expression differences in 20 tissues have been assessed by qRT-PCR only for *XPG* and its two splice variants with residual repair function (IsoV and VI), whereas IsoVI also displayed dominant negative effects in WT cells (see Figures 4C and 5C). Notably, we could observe that individuals with a high expression of isoform VI showed a low expression level of the primary transcript, implicating a dominant negative effect of this variant on flXPG expression. As a future perspective, the quantification of functional splice variants of NER components, e.g. XPG or XPF, in certain high-risk cancer populations (even for different cancer types and tumor entities) could be applied to investigate this variants as prognostic marker for beneficial or negative influences of cancer development, disease outcome, therapeutic responses, and side effects. In summary, we present functionally active, alternatively spliced mRNA variants of XP genes as possible useful tools to estimate cancer susceptibility, responses to chemotherapeutics and therapeutic success [6–8, 69].

## MATERIALS AND METHODS

### Cell culture

WT immortalized MRC5Vi cells were kindly provided by Sarah Sertic (University of Milan, Department of Life Sciences, Milan, Italy). Cells were cultivated and passaged as previously described [13, 14]. For antibiotic selection cells were treated with 600 µg/ml G418 (neomycine) (Sigma-Aldrich, Munich, DE). Primary human fibroblasts from healthy donors were established from skin punch biopsies (WT = male, age 53) in our laboratory. XP20BE cells were purchased from the Coriell Cell Repository (Camden, NJ, USA), while HeLa cells were obtained from ATCC (Manassas, VA, USA). Experiments were conducted in accordance with the Declaration of Helsinki principles, as approved by the University’s ethics committee. Written informed consent was obtained from the donor.

### Cloning

All until now, physiologically occurring spontaneous mRNA splice variants (see Figure 1) of the *XPF* (XPF-201, XPF-003, XPF-202) and *XPG* (XPG IsoII – VI, XPG-201 and XPG-202) genes were amplified from WT MRC5Vi mRNA and cloned into different expression plasmids: pcDNA3.1(+) (for functional testing), pcDNA3.1(-)mycHisA2 (for protein level analyses), and pcDNA3.1 (+)eGFP (C-terminal, for subcellular localization studies). mRNA was isolated from WT MRC5Vi using the RNeasy Kit from Qiagen. Obligatory DNase digestion of RNA samples was performed for 30 minutes at room temperature using the RNase free DNase Set (Qiagen, Hilden, DE) and 1µg of total RNA was used for cDNA synthesis (Revert Aid H Minus Kit, Thermo Fisher Scientific, Waltham, MA, USA) according to manufacturer’s instructions. Splice variants as well as full-length sequences were amplified from cDNA via PCR performed in the Arktik Thermal Cycler (Thermo Fisher Scientific, Waltham, MA, USA). Phusion polymerase (Thermo Fisher Scientific, Waltham, MA, USA) was used according to manufacturer’s instructions. Specifically designed primer pairs were utilized for amplification of the different splice variants and are listed in Table 1. Amplification products of the right size were purified by gel-extraction (Wizard Gel extraction and PCR Clean Up kit, Promega, Mannheim, DE). Restriction sites for specific restriction enzymes were added by a second PCR reaction of the purified fragments and afterwards precipitated with ethanol under high salt conditions. Amplification fragments as well as the target vector were digested with appropriate restriction enzymes (listed in Table 2) according to manufacturer’s protocol (NEB, Ipswich, MA, USA). Ligation was performed in a 3:1 (fragment : vector) ratio using a T4 Ligase (Thermo Fisher Scientific, Waltham, MA, USA). Constructs were transformed in chemically competent *DH5α* (E.coli) cells and isolated from single clones grown on LB Agar plates using the NucleoBond Mini plasmid kit (Machery + Nagel, Düren, DE). Sequences were verified by Sanger sequencing using the BigDye^®^ Terminator v3.1 Cycle Sequencing Kit (Applied Biosystems, Foster City, CA, USA) as described in Schäfer *et al.* [70] (primer see Table 1).

**Table 1:** Oligonucleotides used for cloning, sequencing, and qRT-PCR

Gene	Oligonucleotide
**Amplification primer XPF/XPG and splice variants**	
XPF_fwd	5′- ATGGAGTCAGGGCAGCC -3’
XPF_rev	5′- TCACTTTTTCCCTTTTCCTTTTGA -3’
XPF_rev_w/oStop	5′- CTTTTTCCCTTTTCCTTTTGATAC -3’
XPF201_fwd	5′- ATGGCGCCGCTGCTGGA -3’
XPF003_rev	5′- TTAACCCCACAAGATACCTTCCC -3’
XPF003_rev_w/oStop	5′- ACCCCACAAGATACCTTCCCCT -3’
XPG_fwd	5′- GTGCAGTCCGTCGTAGAAG -3’
XPG_rev	5′- CATTACAAATGGCTGTCATAAC -3’
XP20BE allele_fwd	5′- GTGCAGTCCGTCGTAGAAG -3’
XP20BE allele_rev	5′- AACTTGGGTAAGACTGGGTAG -3’
XPG IsoII_fwd	5′- GTGCAGTCCGTCGTAGAAG -3’
XPG IsoII_rev	5′- CTTTTTTAAAACTTCATCTCTAACACG -3’
XPG IsoIII_fwd	5′- GTGCAGTCCGTCGTAGAAG -3’
XPG IsoIII_rev	5′- CTAGGGCTGCAGCAGAG -3’
XPG IsoIV_fwd	5′- GTGCAGTCCGTCGTAGAAG -3’
XPG IsoIV_rev	5′- GTTACGGTATTACCAAATTAATATC -3’
XPG IsoV_fwd	5′- GTGCAGTCCGTCGTAGAAG -3’
XPG IsoV_rev	5′- GGAAATCCTACCGTTCCAG -3’
XPG IsoVI_fwd	5′- GTGCAGTCCGTCGTAGAAG -3’
XPG IsoVI_rev	5′- GTTACGGTATTACCAAATTAATATC -3’
XPG201_fwd	5′- GAAAGAATGAATCAAAAACAAGC -3’
XPG201_rev	5′- CATTACAAATGGCTGTCATAAC -3’
XPG202_fwd	5′- GAAACAGATAGAGTTGCAACTTG -3’
XPG202_rev	5′- CATTACAAATGGCTGTCATAAC -3’
Sequencing primer	
M13	5′- TAGAAGGCACAGTCGAG -3’
T7	5′- TAATACGACTCACTATAGGG -3’
XPFseq_exon1_fwd	5′- ATGGAGTCAGGGCAGC -3’
XPFseq_exon4_fwd	5′- GTTCCATGTAGCAGTAAACT -3’
XPFseq_exon7_fwd	5′- CAAGTGATGACCGAACATGT -3’
XPFseq_exon9_rev	5′- TCACTTTTTCCCTTTTCCT -3’
XPFseq_exon3_rev	5′- CTTGGCCACAGATACAGTT -3’
XPFseq_exon8_rev	5′- GCTTGGCCACAGATACAGT -3’
XPGseq1_fwd	5′- ACCTCTATGTTTTGCCTCCTT -3’
XPGseq2_fwd	5′- CAACATTCAGGACACATCCG -3’
XPGseq3_fwd	5′- CACTTCTGCAACTGTCTTAGC -3
XPGseq4_fwd	5′- CCACAGACTCAGTTCCAAA -3’
XPGseq5_fwd	5′- GAAGCCTTTCCGATAAGTGAT -3’
XPGseq6_fwd	5′- CTCGGAAGAAAGTGAATCTG -3’
XPGseq7_fwd	5′- ACAACTCTGAGAGGGACGAC -3
XPGseq8_fwd	5′- GGATGTAGGGAATGCCGAAC -3’
XPGseq9_fwd	5′- CTGTCTTCGTTCTGTTCCAG -3
CMVpromoter _fwd	5′- CTGCTTAGGGTTAGGCGTTTTGCGCT -3’
**Oligonucleotides for quantitative real-time PCR purchased from Qiagen, Hilden GER** **Gene Order number**	
GAPDH	QT00079247
**Oligonucleotides for quantitative real-time PCR**	
XPG_fwd	5’- GGATCTTCAAGTGAACATGCTGAA -3’
XPG_rev	5’- TGCGAATCTGAAGCACTGGT -3’
XPGIsoV_fwd	5‘- GAATCTGCAGGCCAGGATTT -3‘
XPGIsoV_rev	5’- CCTACCGTTCCAGATTTCTACAAAA -3
XPGIsoVI_fwd	5’- GAGCCACAGGAAGCTGAGAAA -3‘
XPGIsoVI_rev	5’- CAGCAAGAAGTCGAAACACAATG-3‘

For cloning into pcDNA3.1(+) (KpnI (5’- ttaGGTACC -3’), XbaI (5’- ttaTCTAGA -3’), pcDNA3.1(-)mycHisA2 (XbaI, KpnI), and pcDNA3.1(+)eGFP (KpnI, XbaI) respective restriction recognition sequences were added.

**Table 2:** Restriction enzymes

Description	Manufacturer
KpnI 10u/µl	Thermo Fisher Scientific, Waltham, MA, USA
PvuI	Thermo Fisher Scientific, Waltham, MA, USA
XbaI 10u/µll	Thermo Fisher Scientific, Waltham, MA, USA

### Generation of stable cell lines

Stable cell lines overexpressing the full-length protein or respective splice variants were generated via spontaneous integration of a linearized expression plasmid into MRC5Vi WT cells. An expression plasmid coding for the full-length XPG/XPF protein or one of the splice variants, e.g. pcDNA3.1 (+)XPF, was linearized using the PvuI restriction enzyme (Thermo Fisher Scientific, Waltham, MA, USA) according to manufacturer’s instructions. Successful linearization was analyzed on an agarose gel and the linearized plasmids were purified by ethanol precipitation. Afterwards, the linearized plasmids were transiently transfected into MRC5Vi WT cells seeded on 10mm cell culture dishes and positive clones were selected using G418. Single cell clones were established by serial dilution and checked for plasmid integration via PCR using specific primer pairs for the CMV (cytomegalo virus)-promoter and the integrated gene (see Table 1), as well as immunoblot analyses.

### Immunoblot analyses

Whole protein lysates were extracted from cell pellets, washed twice using 10ml PBS, resuspended in PBS containing PMSF and complete proteinase stop. Cells were disrupted by rotational freezing in liquid nitrogen followed by thawing on ice for three times and sedimented by centrifugation (10 min, 14 000 rpm, 4°C). The supernatant was transferred into a new reaction tube to get rid of cell debris. Lysates were adjusted to the desired concentration by mixing with 3% SDS splitting buffer and boiling at 95°C for 10min. Equal amounts of protein extracts (50–100 µg) were loaded onto the gels. SDS-PAGE and immunoblotting were performed as described before [14] using an anti-XPF mouse monoclonal antibody clone 3F2/3 (Santa Cruz, Dallas, TX, USA), an anti-β-actin mouse monoclonal clone AC-74 (Sigma-Aldrich, Munich, DE) or two different XPG antibodies (Abcam, Cambridge, UK; Bethyl, Montgomery, TX, USA) diluted 1:250, 1:5000, 1:250, and 1:1000 in blocking solution, respectively.

### Reporter gene assays

Analyses of NER, as well as ICL repair capacity and complementation with WT full-length XPF or XPG, as well as the respective splice variants was assessed via HCR performed according to the descriptions in Lehmann *et al.* [14] after 48 h for XPF in immortalized MRC5Vi cells and 72 h for XPG in primary patients or WT fibroblasts. Liposomal transient transfections were carried out in a 24-well format (Greiner Bio-One, Kremsmünster, AUT) using Attractene transfection reagent (Qiagen, Hilden, DE) according to the manufacturer’s instructions. Unirradiated Renilla luciferase was used for normalization. Luminescence was measured 48 h or 72 h after transfection with the GloMax^®^ Discover Multimode Detection System (Promega). The relative repair capability [%] was calculated as the quotient of treated cells to untreated firefly/Renilla ratios. Cells were treated with either UVC (750–1000J/m^2^), with diammineplatinum(II) dichloride (CP) (Sigma-Aldrich, Munich, DE) in a molecular ratio of 1:20 or 1:40 (vector:CP) or with 4,5’,8-trimethylpsoralen (TMP) (Sigma Aldrich, Munich, DE) (1:25 (primary cells) or 1:50 (immortalized cells) (vector:TMP)) followed by irradiation with 1 J/cm^2^ UVA. A batch treatment of plasmids was done and used for all respective experiments. The assay was repeated at least four times in triplicates.

### qRT PCR

Specific primer pairs for isoforms as well as for WT *XPG* were utilized to analyze RNA expression in 20 different healthy human tissues obtained from Ambion^®^ (product discontinued). cDNA was prepared from RNA samples and quantified as described in [14]. Specific primer pairs binding to the intronic regions of the isoforms were applied (see Table 1).

## Abbreviations

6–4PPs: 6–4photoproducts
aa: amino acids
CPDs: cyclobutane pyrimidine dimers
CP: *cis*-diammineplatinum(II) dichloride
CS: Cockayne syndrome
fl: full-length
GGR: global genome repair
HCR: host cell reactivation
(HhH)_2_: Helix-hairpin-Helix motif
ICL: interstrand crosslink
KO: knockout
NER: nucleotide excision repair
NLS: nuclear localization signal
TCR: transcription coupled repair
TFIIH: transcription factor IIH
TMP: 4,5’,8-trimethylpsoralen
WT: wildtype
XP: xeroderma pigmentosum
